# Tapping Force Encodes Metrical Aspects of Rhythm

**DOI:** 10.3389/fnhum.2021.633956

**Published:** 2021-04-22

**Authors:** Alessandro Benedetto, Gabriel Baud-Bovy

**Affiliations:** ^1^Department of Translational Research, University of Pisa, Pisa, Italy; ^2^Robotics, Brain and Cognitive Science Unit, Italian Institute of Technology, Genoa, Italy; ^3^Faculty of Psychology, Vita-Salute San Raffaele University, Milan, Italy

**Keywords:** beat perception, metrical coding, rhythm perception and production, music perception, action and perception, force

## Abstract

Humans possess the ability to extract highly organized perceptual structures from sequences of temporal stimuli. For instance, we can organize specific rhythmical patterns into hierarchical, or metrical, systems. Despite the evidence of a fundamental influence of the motor system in achieving this skill, few studies have attempted to investigate the organization of our motor representation of rhythm. To this aim, we studied—in musicians and non-musicians—the ability to perceive and reproduce different rhythms. In a first experiment participants performed a temporal order-judgment task, for rhythmical sequences presented via auditory or tactile modality. In a second experiment, they were asked to reproduce the same rhythmic sequences, while their tapping force and timing were recorded. We demonstrate that tapping force encodes the metrical aspect of the rhythm, and the strength of the coding correlates with the individual’s perceptual accuracy. We suggest that the similarity between perception and tapping-force organization indicates a common representation of rhythm, shared between the perceptual and motor systems.

## Introduction

When listening to music, people often perceive a certain regularity in the auditory temporal pattern, emerging from the presence of perceptual accents at specific intervals known as “beats.” Our ability to extract rhythmic patterns from temporal events crucially depends on several factors, including the temporal context (Lenc et al., 2020) and cultural environment (Jacoby and McDermott, 2017; London et al., 2017; van der Weij et al., 2017), and this has been widely explored over the years (Grondin, 2010; McAuley, 2010). One of the most influential ideas in time perception research is that humans possess an internal clock mechanism by which we measure and represent time (Treisman, 1963; Treisman et al., 1990). It has been proposed that this clock can synchronize with external events (e.g., sounds) and generates perceptual accents according to a specific set of rules (Povel and Okkerman, 1981; Essens and Povel, 1985). Based on these perceptual rules we can generate *simple rhythms* (i.e., sequences with equally spaced perceptual accents); alternatively, we can generate *complex rhythms* (i.e., sequences with unequally spaced perceptual accents (London, 1995)). The ability to extract a temporal meter from rhythms relies on musical training and on musical exposure to those rhythms (Cameron et al., 2015; London et al., 2017; van der Weij et al., 2017; Bouwer et al., 2018; Nave-Blodgett et al., 2021). This means that certain rhythms may induce, or favor (particularly in participants not previously exposed to those rhythms), perceptual strategies based on non-metrical rules, such as chunking and serial grouping rules (non-metrical coding) (Grahn and Brett, 2007; Grube and Griffiths, 2009; Bouwer et al., 2018).

Hierarchical (metrical) percepts show a vertical organization, with inequalities between the elements of the structure; conversely, non-hierarchical (non-metrical) percepts possess a horizontal organization, with perceptual equalities between the members of the structure. Interestingly, metrical encoding of rhythm is generally accompanied by better performance in both discrimination/perceptual and reproduction/motor tasks than non-metrical encoding (Semjen and Vos, 2002; Patel et al., 2005; Phillips-Silver and Trainor, 2005; Grube and Griffiths, 2009), suggesting that meter perception is a sensorimotor phenomenon (Todd and Lee, 2015). Indeed, moving with the beat improves time perception (Manning and Schutz, 2013), metrical encoding of rhythm can be biased by movements (Phillips-Silver and Trainor, 2005, 2008), and specific activations from the motor system (including basal ganglia, premotor cortex, and supplementary motor area) have been reported during perception of metrical rhythms (Zatorre et al., 2007; Chen et al.,2008a,b; Bengtsson et al., 2009; Grahn and Rowe, 2009; Patel and Iversen, 2014). Furthermore, several studies involving transcranial magnetic stimulation (TMS) demonstrated that motor cortex excitability is directly influenced by the groove of the music. For instance, high-groove music (i.e., music easily inducing a metric encoding) modules corticospinal excitability (Stupacher et al., 2013), and TMS pulses delivered synchronously with the beat for metrically strong sequences generates greater motor-evoked potential responses than for metrically weak sequences (Cameron et al., 2012). In addition, it has been suggested that musicians might possess an effective and automatic internal motor simulation related to beat perception (Su and Poppel, 2012), directly modulating primary motor cortex excitability (Stupacher et al., 2013).

Crucially, beat perception is known to generate a spontaneous synchronization between the perceptual pulses and our movements (Drake et al., 2000; Grahn and Brett, 2007; Repp and Su, 2013; Burger et al., 2014), and different metrical levels are embodied within the whole body (Burger et al., 2014, 2018), suggesting the presence of a common hierarchical organization of the sensory and the motor system during metrical encoding of rhythmic sequences. This sensorimotor link is consistent with the hypothesis of a common encoding strategy shared between the motor and the perceptual systems, or a shared sensorimotor representation of time (Morillon et al., 2014; Morillon and Baillet, 2017; Benedetto et al., 2020). However, this view is challenged by the evidence of a strong auditory vs. visual advantage in sensorimotor synchronization and beat perception (Bartlett and Bartlett, 1959; Patel et al., 2005; Grahn, 2012b), leading to the hypothesis of a special auditory-motor specialization. Tactile modality is certainly the best candidate to compare with auditory: not only do both auditory and tactile information consist of vibrations, but there are profound and early interferences between the two systems (Von Bekesy, 1959; Caetano and Jousmaki, 2006). Nevertheless, only a few studies have investigated rhythm perception and sensorimotor synchronization in the tactile modality, reporting the emergence of beat perception also for this modality (Brochard et al., 2008; Ammirante et al., 2016; Gilmore et al., 2018). However, the hypothesis of an enhanced auditory-motor coupling for rhythmic processing cannot be dismissed (Ammirante et al., 2016).

Grahn (2009) previously investigated finger-tap velocity (an indirect measure of tapping force) during the reproduction of simple rhythms, auditorily presented. The author found that mean velocity was higher on taps presented on the beat than on other taps (Grahn, 2009), suggesting that participants spontaneously organize their motor output according to the metric structure of the percept. However, the internal hierarchy of a metrical sequence might reveal additional levels, and the extent to which force organization mimics this highly structured perceptual hierarchy is currently unknown.

We aimed, here, to provide a clearer picture of the tapping-force organization during rhythm reproduction. First, we assessed the individual perceptual abilities in estimating the temporal order judgments of rhythms varying in complexity in a group of musicians and non-musicians. Secondly, we investigated the fine internal motor representation of the rhythmic sequences (tapping force) during the reproduction of these sequences. To test for the generalizability of the results, rhythms were presented through auditory or tactile modality. The rationale was to investigate if metrical organization of forces (if any) could be driven only by auditory stimulation (as hypothesized by the auditory-motor enhancement hypothesis), or if it generalizes for a different modality.

To summarize the results, we found that the perceptual organization of the rhythm fits the force profile of the finger-taps during its reproduction, irrespective of the modality of stimulation: (*i*) we confirmed the presence of a generalized difference in force for taps presented on the beat vs. other taps; (*ii*) we found that this difference was not modality specific, being present also following a tactile stimulation; (*iii*) we found that the tapping force profile showed a difference between the metrical elements falling on the strong vs. medium elements of the sequence, indicating the presence of a fine-graded and hierarchical motor representation of the rhythm; (*iv*) finally, we show that the amount of *metricality* in the tapping-force, correlates with the perceptual precision, corroborating the idea of a shared and a-modal sensorimotor representation of rhythm.

## Materials and Methods

### Participants

17 volunteers (including one author; age mean ± standard deviation: 23.3 ± 1.6, three women) participated in the study (15 right-handed). We selected participants on the basis of their years of institutional western musical training (music high school and/or conservatory of music in Italy), resulting in eight musicians (age: 22.5 ± 2.2; with at least 5 years of musical training: 7 ± 2; one woman) and nine non-musicians (age 23.9 ± 0.8; no institutional musical training; two women). Musicians played piano (1), bass (1), percussion (2), clarinet (2), trumpet (1), bassoon (1). All the participants performed the temporal-order task (experiment 1), 15 of those were additionally tested for the reproduction task (including one author; eight musicians, seven non-musicians; experiment 2). The studies were reviewed and approved by the local ethics committee (Comitato Etico per la Sperimentazione con l’Essere Umano della ASL 3 di Genova).

### Experimental Setup

The experimental setup was composed of headphones, a tactile stimulator, and a flat piezo-transducer to record finger taps. All devices were connected to a DAQ card (NI DAQ USB-6211) which was controlled through custom C# software run on a PC. The tension of the DAQ analogical output ports controlling the audio or tactile stimuli was updated at a high sampling rate (20 kHz). Note that the output vector that defined the stimulus was computed and loaded in memory using the DAQ dedicated driver and API beforehand so that the timing would be exact. In the reproduction task, one analog input port of the DAQ was used to record the tension produced by the flat piezo-transducer that was fixed on the top of a box. Piezoelectric sensors have the characteristic of transforming mechanical energy into electrical energy and are sensitive to the mechanical force applied. For this reason, the piezo-sensor recorded both the timing of the tap and the force applied by the participants for their finger-tapping. The output tension of the piezo was adjusted by mounting a resistor (22 kΩ) in parallel and sampled at 1000 Hz. The synchronization between output and input signals was ensured by using DAQ hardware synchronization mechanisms. The tactile stimulator was based on a speaker from which the cone had been removed. A light custom-designed 3D printed plastic pin was fixed on the voice coil. To deliver the tactile stimulation, the pin moved vertically through a small hole on the top of the box containing the speaker, where the participants rested their finger. The position of the pin was controlled by setting the tension of the DAQ analogical output port (±10 V), which was amplified through a custom-designed current amplifier. The ability of the device to change position quickly and to apply a constant force on the fingertip was checked with a 6 DOF force sensor (Nano 17, ATI) placed just above the tip of the pin.

### Stimuli and Procedures

The present study comprised two experiments. In experiment 1, participants performed a temporal-order judgment task; in experiment 2 they completed a non-isochronous continuous finger-tapping (reproduction task). The stimuli consisted of six sequences; half of the stimuli were simple sequences and the other half complex sequences. Simple rhythms were defined as sequences with equally spaced perceptual accents in time (e.g., a perceptual accent every 400 ms), and complex rhythms as sequences with unequally spaced perceptual accents in time (e.g., the delay between accents varied between 400 and 600 ms) (Povel and Okkerman, 1981; Essens and Povel, 1985). Each stimulus was composed of five elements, that could have either a single (1) or double (2) unit duration (determining the *speed* of the sequence), and the same intensity. The unit duration varied between 200, 223, 246, and 269 ms. The three simple sequences were: 21122, 22112, 22211 (*simple rhythm*). The three complex sequences were: 11122, 21112, 22111 (*complex rhythm*). The intervals between each element of the sequence were obtained by shortening each element by 50 ms. Within each session, the duration of the unit varied randomly across trials to avoid learning effects and keep participants attentive to each rhythm. The auditory and tactile rhythmic sequences were presented block-wise. The auditory rhythmic elements were pure tone of 440 Hz (output tension amplitude: ± 0.05 V). The tactile sequences consisted of stimulation of the index fingertip of the non-dominant hand by a custom tactile stimulator. For both conditions, an auditory white noise (0.03 V) was superimposed onto the signal during the whole trial duration to assure acoustic isolation from the tactile stimulator and from the finger tapping.

### Experiment 1: Temporal-Order Task

**FIGURE 1:**
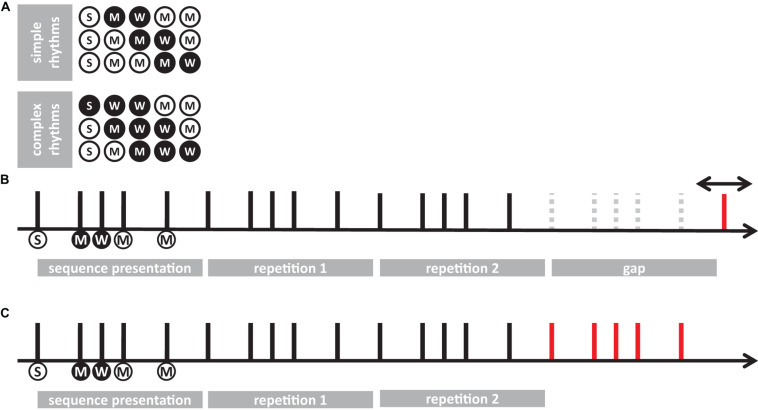
A schematic of the experimental procedures. **(A)** representation of simple and complex rhythms adopted in the present study. Each circle represents a unit of the sequence. White and black circles denote long and short elements, respectively. Letters in the circles indicate the predicted level of perceptual accent for each element: “S” for strong, “M” for medium; “W” for weak. **(B)** trial example from temporal-order task (experiment 1). Each vertical line represents the onset of the element for a given sequence (e.g., 21122). Circles below the lines report the length and the perceptual accent for each element (following the symbol conventions from **A**). The sequence was presented 3 times in succession, then—after an interval gap of the duration of the sequence plus a random jitter—a probe was presented (red line). Participants had to report whether the probe was presented before or after the predicted onset of the imagined 5th sequence. **(C)** Example of reproduction task (experiment 2). After presenting the sequence three times, participants were asked to continue the reproduction (red line) for about 20 s, by tapping with their finger on a piezo-electric sensor.

Each trial started with a rhythmic sequence (auditory or tactile) presented in a continuous loop for a total of three times. Participants were asked to imagine two following repetitions and to decide whether the beginning of the last repetition preceded or followed a probe (200 ms of duration) that occurred around the time of its ideal onset (Figure 1B). The probe preceded or followed the ideal onset of the last repetition by a 5, 10, 20, or 40% of the global duration of the sequence. After each trial, participants verbally reported whether the probe preceded or followed the ideal onset of the sequence. The probe consisted of an 800 Hz pure tone for the auditory condition and a tactile stimulation for the tactile condition. Each modality (auditory or tactile) and rhythm complexity (simple or complex) was tested in separate blocks. Each sequence was combined with the four possible speeds (determined by the unit duration), and all the possible time lags, which yielded 96 trials per condition. The order of presentation of the rhythmic sequences and tempi within each condition was randomized. The experiment lasted approximately 80 min and was divided in two separate sessions.

### Experiment 2: Reproduction Task

Experiment 2 used the same stimuli as in experiment 1. A sequence was presented in a continuous loop for a total of three times. Participants were instructed to start the reproduction of the perceived rhythm after the end of the last repetition, and to reproduce the sequence until the end of the trial, which lasted 25 s (Figure 1C). The end of the trial was signaled by the stop of the white noise delivered via headphones. Participants were asked to tap with the index fingertip of their dominant hand on a piezoelectric transducer. Each modality (auditory or tactile) and rhythm complexity (simple or complex) was tested in separate blocks. Each stimulus was presented once for each speed (unit duration), which yielded 12 trials per condition. All four conditions were acquired during a unique recording session.

### Data Analysis

#### Experiment 1: Temporal-Order Task

A cumulative gaussian function was fitted to the participants’ responses as a function of the probe delay expressed as a percentage of the rhythm duration. For each participant, sensory modality (auditory or tactile) and rhythm complexity (simple or complex), we estimated the point-of-subjective-simultaneity (PSS) and the just-notable-difference (JND). PSS measures a possible temporal shift of the perceived rhythm onset while JND measures the temporal variability of the perceived onset. Mixed-design repeated measure ANOVA on PSS and JND was run to estimate the main effects and the interaction of a full model (2 rhythm complexities × 2 modalities × 2 expertise, with rhythm complexity and modality as within factors, and expertise as between-subject effect). To satisfy the conditions of the ANOVA and power analysis (homoscedasticity and homogeneity of variance between groups), PSS and JND were transformed with a cubic-root transformation. For all statistically significant effects, we reported the generalized eta-squared $\eta_{G}^{2}$ with expertise as a measured variable (Olejnik and Algina, 2003).

For all analyses, we conducted a power analysis that combined elements of *a priori* and *post hoc* analysis. For all tests, the unstandardized effect size was determined on *a priori* ground, as the smallest effect that we considered to be meaningful or interesting. The variance-covariance structure was determined from the results of the experiments. In particular, we retained that a difference of 5% for the PSS and the JND was a meaningful difference for all effects and used the results of the experiments to estimate all relevant sources of variance. Given the sample sizes of this study, the probability of detecting a 5% temporal shift difference between the two groups was 51% for the PSS and 44% for the JND analysis. However, the power of detecting such a 5% difference was above 86% for all other effects in the PSS analysis, including interactions with the between-subject group factor, and above 99% for all effects in the JND analysis.

To deal with the unbalance between the two groups, all power analyses were conducted in R with simulations (Arnold et al., 2011). First, we used linear mixed-effect models to identify the variance-covariance structure for each variable (Bates et al., 2015; Matuschek et al., 2017). In general, the identified variance-covariance structure included a random intercept for the subject and, in several cases, an uncorrelated random slope for one within-subject factor. More complex variance-covariance structures were not supported by the data. Note that the linear mixed-effect model (LME) included the same fixed effects as the corresponding mixed-effect repeated-measure ANOVA and that the *p*-values of both analyses were very similar in all cases. Second, we used the LME model with the variance-covariance structure identified in the previous step to simulate datasets. The fixed effects of these LME models were defined so that all main effects and interactions would include a meaningful difference (see above). Finally, we estimated the power by analyzing each simulated dataset with a mixed-effect repeated-measure ANOVA as described above and by tallying the number of times each effect was statistically significant. In general, these analyses indicated that the sample size was sufficient to detect reliably a meaningful difference if it existed with the exception of the expertise main effect. This result reflects the fact that the power for the main effect corresponding to a between-subject factor in a split-plot design is markedly lower than the power of the within-subject factors and/or all their interactions (including the interactions between between-subject and within-subject factors) (Bradley and Russell, 1998). Note that this approach is different from standard *post hoc* power analysis where the effect size is determined by the actual results. Provided that the power is large enough, it ensures that a meaningful effect would be statistically significant. Accordingly, it also ensures that a non-statistically significant test is not due to lack of power but to an effect size that is of little interest. Still, the results of the simulations should be interpreted cautiously as it is not possible to exclude the possibility that the simulated datasets underestimate or misrepresent the variance in the actual populations given the limited sample size of the study.

In addition to frequentist statistics, we also implemented a Bayesian analysis for the repeated measure ANOVA (JASP version 0.9.1.0), and for *t*-tests. Bayesian analysis of effects in the ANOVA were computed across matched models. We report the change from prior to posterior inclusion odds (BF_*inclusion*_). The JZS Bayes Factor (BF10) was estimated for paired *t*-tests with a default scale factor of 0.707 (Liang et al., 2008; Rouder et al., 2009).

### Experiment 2: Reproduction Task

#### Reproduction Quality

Given the circular nature of the tapping behavior, the tapping performance was assessed using circular measures (Della Bella et al., 2017). For each trial, we defined an expected sequence to be reproduced (i.e., the presented sequence), that we compared with the observed one. Each recorded tap was categorized as long (double duration) or short (single duration), according to the expected order of long and short elements. Within each category, we discarded outliers, defined as taps farther away than 2 standard deviations from the average observed delay (about 4% of total taps). We transformed each tap-delay into a phase (φ_*k*_), according to its expected delay with the formula:

(1) $$\varphi_{k}=2\,\pi \,\frac{x_{k}}{\hat{x}{}_{k}}$$

where *x_k* is the observed, and ${\hat{x}}_{k}$ is the expected delay for the tap k. We computed the vector length (*R*, synchronization precision) and the angle (Ψ, synchronization accuracy) of the resulting distribution as:

(2) $$R=\frac{\left|\sum_{k=1}^{N}e^{i\,\varphi_{k}}\right|}{N}$$

and

(3) $$\Psi =\arg\,\left(\sum_{k=1}^{N}e^{i\,\varphi_{k}}\right)$$

In order to compensate for possible missing taps, the procedure was run for each possible expected sequence obtainable from the original one (e.g., from the sequence “22112”: “22112,” “21122,” “11222,” “12221,” “22211”). Thus, for each trial we ended up with five couples of measures (two for each tested sequence). The measures referring to the sequence with the higher synchronization precision were kept for further analysis. Trials with a poor synchronization precision (below 0.5) were discarded from further analysis (about 5% of trials). The procedure resulted in two indexes of reproduction quality: (i) synchronization precision (*R*), and (ii) synchronization accuracy (Ψ). Synchronization precision measured the participants’ precision in reproducing the sequence, and its values were bounded between 0 (minimal precision) to 1 (maximal precision). To meet the ANOVA assumptions about the normal distribution of residuals, the synchronization precision was Fisher-transformed (*z*) using the inverse hyperbolic tangent of the vector length (*R*), as:

(4) $$R_{z}=\frac{1}{2}\,\ln\,\left(\frac{1+R}{1-R}\right)$$

Synchronization accuracy measured the participants’ accuracy in reproducing the sequence, with values reported in radians and bounded between −π and π, where values closer to 0 indicate maximal accuracy and closer to π indicate minimal accuracy. For the two indexes, we evaluated the effects of rhythm complexity, expertise and modality by a mixed-design repeated measure ANOVA (2 rhythm complexities × 2 modalities × 2 expertise, with rhythm complexity and modality as within factors, and expertise as between-subjects effect). Power was computed similarly as in experiment 1, with the smallest meaningful effect size set as 0.1 for synchronization precision, and 0.15° for synchronization accuracy. The probability of detecting a meaningful difference between the two groups was only 9% for synchronization precision and 23% for the synchronization accuracy analysis. For the synchronization precision analysis, power was also low for rhythm complexity (21%) and the interaction between group and rhythm complexity (22%). The power of detecting a meaningful difference in synchronization precision or accuracy was above 87 and 77%, respectively, for all other effects and interactions.

#### Reproduction Force

For each element of each sequence, we predicted—according to metrical encoding rules (Povel and Okkerman, 1981; Essens and Povel, 1985)—the strength of its perceptual accent (corresponding to the hierarchical level of the element in the sequence). This prediction led to a categorization of each element as strong (S), medium (M), and weak (W) (see Figure 1A). Strong elements (S) were defined as those elements falling on the perceptual beat and starting the sequence (i.e., the elements with the higher hierarchical position). Medium elements (M) were defined as elements falling on a perceptual beat but not in a starting position. Finally, weak elements (W) were defined as those elements that did not fall on a perceptual beat.

The reproduction force of each tap was estimated by computing the maximal voltage generated around the time of each tap by the piezo-sensor (within −10 to 50 ms from tap onset). In order to directly compare forces across trials and conditions, they were standardized (z-scored) within each trial, and a repeated measure ANOVA was run to investigate the main effects of modality, rhythm complexity and element (2 modalities × 2 rhythm complexities × 3 elements). The test of Maunchly revealed a violation of sphericity for the factor “element” (Maunchly’s *W* = 0.514; *p* = 0.018). Consequently, a Greenhouse-Geisser sphericity correction was applied to the degrees of freedom of the ANOVA. Power was computed similarly as in experiment 1, with the smallest meaningful effect size set as 0.1. Power was above 94% for all the effects and interactions, except for the element main effect (19%).

We defined an index of metrical coding resulting in the difference between the mean force generated to reproduce the strong and the medium elements of each sequence (*metrical coding index*). This comparison allowed us to investigate further the hierarchical organization of the forces, i.e. the dissimilarities between elements presented on the perceptual beat (note that weak elements were not presented on the perceptual beat).

## Results

### Experiment 1: Temporal-Order Task

**FIGURE 2:**
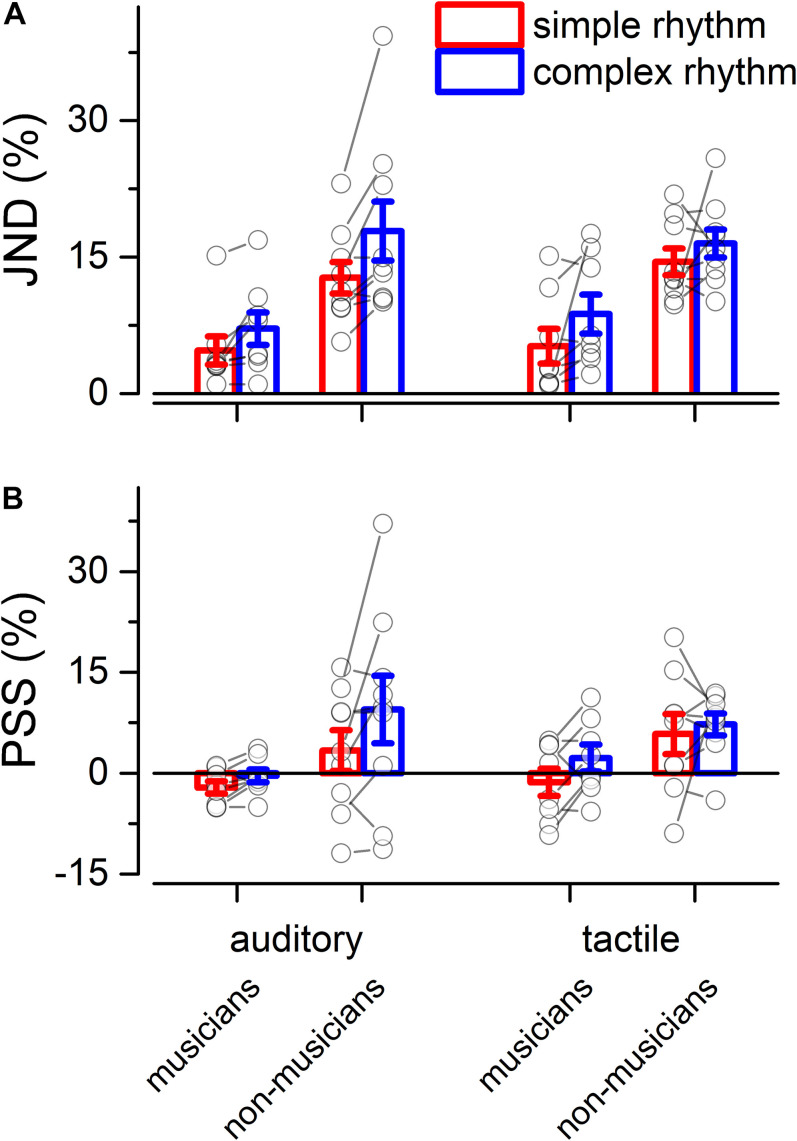
Experiment 1: temporal order judgment task. **(A)** Grand-average just-noticeable-difference (JND) for simple (red) and complex rhythms (blue) for all the modality conditions and expertise. Dots indicate individual data, error bar indicates ± 1 S.E.M. **(B)** Same as **(A)** but for point-of-subjective-simultaneity (PSS).

Separated repeated measure ANOVA analyses were conducted on the transformed JND and PSS to investigate the main effect of rhythm complexity (simple vs. complex), expertise (non-musicians vs. musicians), and modality (auditory vs. tactile). As shown in Figure 2A, the analysis on JND revealed an effect of expertise [*F*(1, 15) = 17.97, *p* < 0.001, $\eta_{G}^{2}$ = 0.43; BF10_*i*__*nclusion*_= 39.41] and rhythm complexity [*F*(1, 15) = 17.31, *p* < 0.001, $\eta_{G}^{2}$ = 0.05; BF10_*i*__*nclusion*_= 227.56]. No other effects or interactions were statistically significant (*p* > 0.05, see Table 1). The PSS revealed a statistically significant effect of expertise [*F*(1, 15) = 5.51, *p* = 0.033, $\eta_{\text{G}}^{2}$ = 0.17; BF10_*i*__*nclusion*_ = 2.32] and rhythm complexity [*F*(1, 15) = 7.30, *p* = 0.01, $\eta_{\text{G}}^{2}$ = 0.034; BF10_*i*__*nclusion*_ = 2.38; see Figure 2B]. No other main effects or interactions were statistically significant (*p* > 0.05, see Table 2). Overall, these results indicate a higher perceptual precision and accuracy for musicians compared to non-musicians, as well as a general perceptual advantage in temporal order judgments for simple sequences compared to complex sequences, independent of the modality of stimulation.

**TABLE 1:** Repeated measure ANOVA—JND (experiment 1).

Effect	Df num.	DF den.	MSE	*F*	$\eta_{G}^{2}$	*P*-value
Expertise	1	15	0.488	17.975	0.469	**<0.001**
Modality	1	15	0.055	0.818	0.005	0.380
Expertise:modality	1	15	0.055	0.006	<0.001	0.938
Complexity	1	15	0.062	17.312	0.098	**<0.001**
Expertise:complexity	1	15	0.062	1.354	0.008	0.263
Modality:complexity	1	15	0.057	0.005	<0.001	0.943
Expertise:modality:complexity	1	15	0.057	1.680	0.009	0.214

**Transformation: x^1/3^*. Bold highlights the statistically significant results (*p* < 0.05).*

**TABLE 2:** Repeated measure ANOVA—PSS (experiment 1).

Effect	Df num.	DF den.	MSE	*F*	$\eta_{G}^{2}$	*P*-value
Expertise	1	15	1.78	5.51	0.175	**0.033**
Modality	1	15	0.75	1.22	0.016	0.286
Expertise:modality	1	15	0.75	0.06	<0.001	0.804
Complexity	1	15	0.27	7.30	0.034	**0.016**
Expertise:complexity	1	15	0.27	0.01	<0.001	0.911
Modality:complexity	1	15	0.27	0.00	<0.001	0.976
Expertise:modality:complexity	1	15	0.27	0.30	0.001	0.590

**Transformation: sign(x)((| x| + 1)^1/3^ − 1). Bold highlights the statistically significant results (*p* < 0.05).**

### Experiment 2: Reproduction Task

#### Reproduction Quality

**FIGURE 3:**
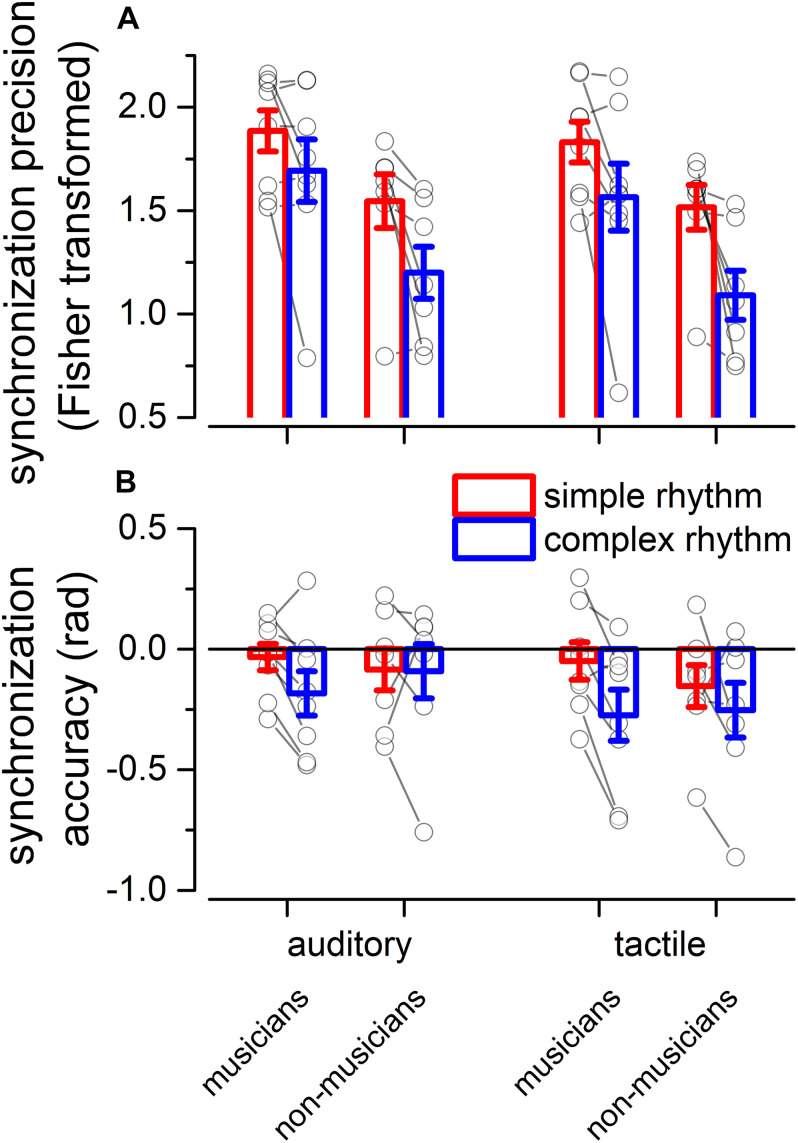
Experiment 2: reproduction quality. **(A)** grand-average synchronization precision for simple (red) and complex rhythms (blue) for all the modality conditions and expertise. Dots indicate individual data, error bar indicates ± 1 S.E.M. **(B)** Same as **(A)** but for synchronization accuracy.

We evaluated two indexes of reproduction quality: *i*) synchronization precision, and *ii*) synchronization accuracy (see section “Materials and Methods” for details). Repeated measure ANOVA on synchronization precision revealed a significant effect of expertise [*F*(1, 13) = 6.99, *p* = 0.02, $\eta_{\text{G}}^{2}$ = 0.23; BF10_*i*__*nclusion*_ = 3.28], modality [*F*(1, 13) = 6.20, *p* = 0.027, $\eta_{\text{G}}^{2}$ = 0.009; BF10_*i*__*nclusion*_ = 0.64] and rhythm complexity [*F*(1, 13) = 13.15, *p* = 0.003, $\eta_{\text{G}}^{2}$ = 0.13; BF10_*i*__*nclusion*_ = 9532.69], and no significant interactions (*p* > 0.05, see Table 3 and Figure 3A). The same analysis performed on the synchronization accuracy revealed a main effect of rhythm complexity [*F*(1, 13) = 5.50, *p* = 0.036, $\eta_{\text{G}}^{2}$ = 0.054; BF10_*i*__*nclusion*_ = 8.97], and no other significant effects or interactions (*p* > 0.05) (see Table 4 and Figure 3B). Overall, the results confirm that complex sequences were generally reproduced with less precision and accuracy then simple sequences.

**TABLE 3:** Repeated measure ANOVA—synchronization precision (experiment 2).

Effect	Df num.	DF den.	MSE	*F*	$\eta_{G}^{2}$	*P*-value
Expertise	1	13	0.35	6.99	0.281	**0.020**
Modality	1	13	0.01	6.20	0.015	**0.027**
Expertise:modality	1	13	0.01	0.11	0.000	0.740
Complexity	1	13	0.10	13.15	0.183	**0.003**
Expertise:complexity	1	13	0.10	0.84	0.014	0.374
Modality:complexity	1	13	0.01	2.19	0.003	0.162
Expertise:modality:complexity	1	13	0.01	0.00	0.000	0.958

**Transformation: atanh(x). Bold highlights the statistically significant results (*p* < 0.05).**

**TABLE 4:** Repeated measure ANOVA—synchronization accuracy (experiment 2).

Effect	Df num.	DF den.	MSE	*F*	$\eta_{G}^{2}$	*P*-value
Expertise	1	13	0.19	0.00	0.000	0.928
Modality	1	13	0.02	4.34	0.028	0.057
Expertise:modality	1	13	0.02	0.58	0.003	0.457
Complexity	1	13	0.03	5.49	0.057	**0.035**
Expertise:complexity	1	13	0.03	1.70	0.018	0.213
Modality:complexity	1	13	0.01	2.32	0.007	0.151
Expertise:modality:complexity	1	13	0.01	0.02	0.000	0.873
						

*Bold highlights the statistically significant results (*p* < 0.05).*

#### Reproduction Force

**FIGURE 4:**
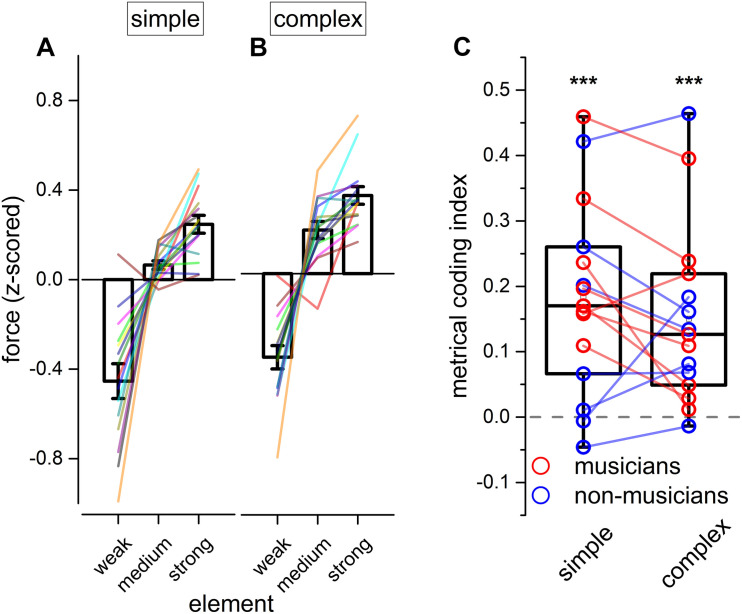
Experiment 2: reproduction force. **(A,B)** Grand-average force profile of the finger-tapping for the different elements of a simple **(A)** and complex sequence **(B)**. Error bar indicates ± 1 S.E.M. Colored lines show single participants. **(C)** Boxplot showing the metrical coding index distribution for both simple and complex sequences. Dots indicate individual data (musicians are marked in red, and non-musicians in blue); error bar indicates confidence intervals (95 %ile). Asterisks mark the statistical significance (*p*-value): *** <0.001.

With the goal of investigating the internal motor organization of rhythm, we focused on the force aspects of the reproduction (see Figure 4). Repeated measure ANOVA on the applied force revealed a main effect of the element [*F*(1.25, 17.52) = 52.70, *p* < 0.001, $\eta_{\text{G}}^{2}$ = 0.70; BF_*inclusion*_ = 2.874e+43], rhythm complexity [*F*(1, 14) = 45.77, *p* < 0.001, $\eta_{\text{G}}^{2}$ = 0.06; BF_*inclusion*_ = 43.36], and a significant triple interaction between modality, rhythm complexity and element [*F*(1.46, 20.48) = 4.70, *p* = 0.029, $\eta_{\text{G}}^{2}$ = 0.02; BF_*inclusion*_ = 0.81; see Table 5]. Importantly, Bayesian repeated measure ANOVA revealed that the best model was the one including only rhythm complexity and element (BF_*model*_ = 47.78). *Post hoc* comparisons revealed that weak elements were reproduced with less force than medium [*t*(14) = −7.46, p_*holm*_ < 0.01, Cohen’s *d* = −1.92, BF10 = 1.075e+13] and strong elements [*t*(14) = −9.83, p_*holm*_ < 0.001, Cohen’s *d* = −2.54, BF10 = 1.135e+17], and medium elements were reproduced with less force than strong elements [*t*(14) = −2.37, p_*holm*_ = 0.025, Cohen’s *d* = −0.61, BF10 = 2.588e+6]. To summarize, results revealed that rhythm complexity and element modulated the force of the tap. Crucially, a force difference was evident not only between elements presented on the beat (strong and medium elements) vs. elements presented off the beat (weak elements), but also between strong and medium elements (i.e., both elements presented on the perceptual beat but differing in the hierarchy of the sequence; see Figures 4A,B). Although expertise was not included in the ANOVA for sake of simplification, the average force for each musician and non-musician are indicated in Figure 4.

**TABLE 5:** Repeated measure ANOVA—force (experiment 2).

Effect	Df num.	DF den.	MSE	*F*	$\eta_{G}^{2}$	*P*-value
Modality	1	14	0.00	0.25	0.000	0.618
Complexity	1	14	0.01	45.77	0.067	**<0.001**
Element	1.25	17.52	0.24	52.70	0.707	**<0.001**
Modality:complexity	1	14	0.00	0.04	0.000	0.840
Modality:element	1.91	26.84	0.03	0.49	0.004	0.604
Complexity: element	1.19	16.74	0.04	0.11	0.000	0.779
Modality:complexity:element	1.46	20.48	0.02	4.70	0.022	**0.029**

*Bold highlights the statistically significant results (*p* < 0.05).*

To assure that the reported differences in force were not simply due to a physical constraint imposed by the duration of the current element to be reproduced, we replicated the analysis by focusing on strong and medium elements with double unit durations (i.e., the double elements presented on the perceptual beat but differing in the hierarchy of the sequence. The sequence “11122” was discarded from this analysis because of the short duration of the strong element). Results revealed significant effects of rhythm complexity [*F*(1, 14) = 25.59, *p* < 0.001, $\eta_{\text{G}}^{2}$ = 0.17; BF_*inclusion*_ = 8033.50] and element [*F*(1, 14) = 6.01, *p* = 0.028, $\eta_{\text{G}}^{2}$ = 0.1; BF_*inclusion*_ = 78.99], indicating that participants used more force in reproducing the strong element of the sequence as compared to the medium one, and this effect cannot be attributed to the duration of the element that was reproduced.

The temporal gaps between taps might influence the applied force. In order to control for this possibility, we ran a repeated-measure ANOVA on the speed of the sequences (unit duration). If the level of force depends on the temporal gap between taps, we expect an effect of speed (e.g., lower force for faster rhythms, where the gaps are shorter than for slower rhythms). The ANOVA revealed no significant effects or interactions [speed: *F*(3, 42) = 1.06, *p* = 0.374, $\eta_{\text{G}}^{2}$ = 0.07; BF_*inclusion*_ = 0.26], suggesting that the reported effect was driven by the position of the element in the sequence and not by physical constraints.

To summarize the previous findings, we measured the force difference between the strong and the medium elements of each sequence (*metrical coding index*, see Figure 4C). Values above 0 would indicate that the strong element was reproduced with more force than the medium one. In other words, a value above 0 would indicate a coherence between the predicted metrical hierarchy of the element and the force applied in reproducing it and, hence, the presence of metrical coding in the force. Conversely, values around 0 would indicate that tapping force was not coding the metrical aspects of the rhythm. Two-tail paired t-tests were run for simple and complex sequences. Results demonstrated that participants encoded metrical information in force for both simple and complex sequences [simple rhythm: *t*(14) = 4.79, p_*holm*_ < 0.001, BF10 = 114.39; complex rhythm: *t*(14) = 4.29, p_*holm*_ < 0.001, BF10 = 50.32].

**FIGURE 5:**
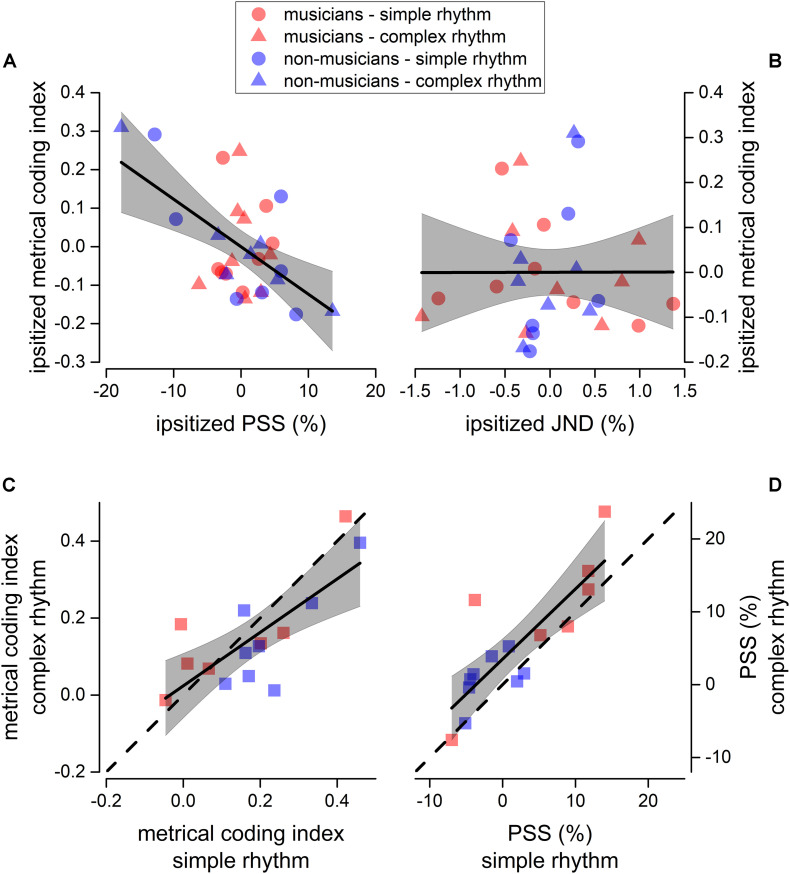
Sensorimotor correlation. Linear regression between ipsitized metrical coding index and ipsitized PSS **(A)**, and between ipsitized metrical coding index and ipsitized JND **(B)**. We plotted a value for each participant (musicians in red, and non-musicians in blue) and rhythm complexity (simple as circles, complex as triangles). Thick lines show the best linear regression of data, gray areas show 95% confidence band. The linear regression revealed a significant negative correlation between metrical coding index and PSS (*p* < 0.05, see section “Materials and Methods for details). **(C,D)** Correlation between metrical coding index for simple (*x*-axis) and complex (*y*-axis) rhythms **(C)** and between PSS for simple (*x*-axis) and complex (*y*-axis) rhythms **(D)**. Dashed lines report the equality line, thick lines show the best linear regression of data, gray areas show 95% confidence band. Red and blue squares indicate musicians and non-musicians, respectively.

It is known that metrical coding favors both rhythm perception and reproduction (Semjen and Vos, 2002; Phillips-Silver and Trainor, 2005; Grahn and Brett, 2007; Chen et al., 2008a; Grube and Griffiths, 2009; Cameron and Grahn, 2014). To investigate this aspect further, we correlated the perceptual performance from experiment 1 (for both JND and PSS) with the *metrical coding index*. To account for possible spurious correlations introduced by differences in experimental conditions and expertise, all the indexes to-be-correlated were mean-centered (*ipsitized*) for rhythm complexity and expertise (e.g., the distribution of the JNDs pertaining to musicians in complex rhythms was centered at 0). Results revealed that the PSS correlated with metrical coding abilities (Pearson’s *r* = −0.56; *p* = 0.001), while JND did not (Pearson’s *r* = 0.002; *p* = 0.99, see Figures 5A,B).

Finally, we found that JND (not shown), PSS, and metrical coding index within participants strongly correlated across rhythm complexities [JND: *F*(1, 13) = 72.4, *p* < 0.001, *r*(15) = 0.92; PSS: *F*(1, 13) = 27.9, *p* < 0.001, *r*(15) = 0.82; metrical coding index: *F*(1, 13) = 17.1, *p* = 0.001, *r*(15) = 0.75; see Figures 5C,D).

## Discussion

Volunteers (musicians and non-musicians) were asked to separately perform an auditory and a tactile temporal-order judgment task (experiment 1), where the experimenter manipulated the complexity of the rhythm. In a second experiment, volunteers were asked to reproduce the same rhythms, and the timing and force of their reproduction was analyzed (experiment 2).

Our results revealed that (*i*) rhythm complexity affects precision and accuracy for both perceptual and reproduction tasks; (*ii*) tapping force was higher for elements presented on the beat than for elements falling outside the beat (or on the weak beat); (*iii*) metrical elements falling on the strong vs. medium beat of the sequence were further differentiated, indicating the presence of a complex hierarchical motor organization; (*iv*) this force difference was not modality specific; (*v*) the sensorimotor organization of rhythms was the same for different modalities of rhythm presentation (auditory or tactile); and finally, (*vi*) the amount of *metricality* in the tapping-force correlates with the individual perceptual accuracy.

Overall, results from experiment 1 and 2 revealed the presence of a general effect of rhythm complexity on sensorimotor precision and accuracy: simple rhythms were perceived and reproduced with more accuracy and precision compared to complex rhythms. There is currently no consensus about the key factors making a rhythm “complex” or “simple” (Grahn, 2012a). We speculate that a main difference between the two types of rhythm might be linked to the number of clocks (or oscillators) needed to univocally predict the pulses generated by the rhythm itself (i.e., the complexity of the predictive model). Subsequent pulses in simple rhythms are perceived at fixed and equal temporal intervals, and a single oscillator could easily model the perceptual distribution of pulses. On the other hand, complex rhythms generate un-equally spaced pulses, that cannot be modeled by a single oscillator, implicating the presence of multiple phase-locked oscillators working together to predict the timing of the perceptual pulses. In this sense, simple predictive models might be advantageous for perception and sensorimotor synchronization as they generally might require less attentional and mnemonic (and generally neuronal) resources, as compared to complex models of prediction.

Although the facilitatory effect of simple rhythms was clearly visible for both groups, we also found that musicians perceived and, to some extent, reproduced complex rhythms better than non-musicians. It is noteworthy that musicians received an institutional western musical training in Italy, which includes the exposure to complex rhythms. For this reason, complex rhythms are considered metrical by most musicians. In contrast, the impact of the subjective experience in shaping rhythm perception is intrinsically more problematic for non-musicians (reporting no institutional music training) despite possible exposure to rhythms and music that might have included complex rhythms. In this respect, it should be noted that the perception of metrical structures in a rhythm strongly relies on subjective experiences and culture (Cameron et al., 2015; London et al., 2017; van der Weij et al., 2017; Bouwer et al., 2018; Nave-Blodgett et al., 2021) and could be shaped by passive exposure to music (Jacoby and McDermott, 2017). For instance, some of the complex rhythms adopted here are common in certain folk music from Balkan, east European, or in jazz music, but almost absent in other cultures (e.g., they are almost absent in the Italian folk music, the state where participants were recruited and tested). Unfortunately, we could not investigate this aspect further, as non-musicians reported no institutional training in music, and we did not evaluate their degree of musical expertise with any other scale.

Grahn (2009) demonstrated that tap velocity (an indirect measure of tapping force) during the reproduction of simple rhythms was higher on taps presented on the beat than on other taps (Grahn, 2009). We replicated this effect showing that the weak elements of a sequence (i.e., those elements not falling on the beat, or falling on sub-division of it) are reproduced with less force than medium and strong elements. Importantly, we extended this result by showing that the strong and medium elements (i.e., elements falling on the beat, but pertaining to different hierarchical levels) were further reproduced with different intensities, indicating the presence of a complex hierarchical coding.

We found here the presence of a comparable hierarchical organization of forces for both simple and complex rhythms. Indeed, a close look at the metrical coding index, revealed that—across participants—it strongly correlated for the different rhythm complexities. This suggests that, irrespective of a general sensorimotor facilitation for simple vs. complex rhythms, participants were able to code both simple and complex sequences adopting a hierarchical organization. Importantly, the correlation between perceptual accuracy and metrical coding suggests the presence of a continuous gradient in which participants with a stronger metrical coding were also more accurate in the perceptual task. It would be interesting, for future research, to investigate the organization of forces on different rhythms (e.g., rhythms with different integer ratios, or purely non-metrical sequences) and directly comparing it for musicians, non-musicians, and cross-culturally.

Metrical coding has been mostly reported in auditory modality, suggesting that this ability might be specific to the auditory system (Bartlett and Bartlett, 1959; Patel et al., 2005; Grahn, 2012b). In line with this hypothesis, a close link between sensorimotor brain activity and auditory temporal predictions or musical imagery has been demonstrated (Morillon et al., 2014; Morillon and Baillet, 2017; Gelding et al., 2019). Motor cortex excitability and coordination is directly influenced by the groove of the auditory stimulation (Wilson and Davey, 2002; Stupacher et al., 2013); however, metrical coding has been recently shown in tactile perception (Brochard et al., 2008; Ammirante et al., 2016; Gilmore et al., 2018), questioning the existence of a special link between beat perception and auditory modality (Ammirante et al., 2016).

Current results revealed no effect of modality in the perceptual task (for both perceptual accuracy and precision) and in reproduction accuracy, but a significant (although weak) effect of modality for reproduction precision. Crucially, the analysis on forces revealed no effect of the modality of stimulation, indicating the presence of a similar hierarchical organization of forces under both stimulation conditions, and further questioning the hypothesis of specific auditory-motor processes for rhythm perception and reproduction.

How might this shared temporal rhythm be orchestrated in the brain? The synchrony between different perceptual systems (or between perception and action) is challenged by the evidence that different processes elaborate the information at different speeds: for instance, time has been shown to vary across sensory modalities and features of the sensory stimulation (Johnston et al., 2006; Kanai et al., 2006; Burr et al., 2011; Harrington et al., 2011; Tomassini et al., 2011). It has been suggested that motor system might play a key role in shaping a unitary sense of time, possibly by synchronizing the dynamics of local processing (Hommel et al., 2001; Benedetto et al., 2018, 2020). This hypothesis is in line with the evidence that time perception and motor timing rely on similar cerebral structures (Schubotz et al., 2000; Nobre and O’Reilly, 2004).

Passive perception of metrical rhythms is known to elicit the activity of several motor areas (Zatorre et al., 2007; Chen et al.,2008a,b; Bengtsson et al., 2009; Grahn and Rowe, 2009; Patel and Iversen, 2014), and it has been suggested that it might involve an internal motor simulation, phase-locked with the pulse of the rhythm (Wilson and Davey, 2002; Su and Poppel, 2012). Manning and Schutz (H2013) demonstrated that timekeeping is improved when participants move along with a rhythm. In their study, participants heard a series of isochronous sounds followed by a short silence and a probe beat, and they were asked to judge whether the timing of the probe was consistent with the timing of the preceding sequence. The authors found an improvement in the perceptual performance when participants tapped along with the beat, remarking on the presence of a beneficial crosstalk between the sensory and the motor system (Manning and Schutz, 2013) and suggesting that perceptual contents and action plans are coded in a common representational medium (Hommel et al., 2001). In line with this hypothesis, our results demonstrated further that the organization of the rhythm is directly encoded in the force profile of the finger-taps, and this effect is independent of the modality of stimulation.

## Conclusion

To sum up, our results show the presence of a hierarchical organization of the forces during finger-tapping to reproduce a rhythm, and this organization mimics the perceptual hierarchical organization of the rhythm itself. We speculate that this effect reflects the presence of a shared representation of rhythm between the motor and the sensory systems, i.e., a supra-modal representation of time shared within the sensorimotor circuit that facilitates both the perception and the reproduction of the temporal sequences.

## Data Availability Statement

Experimental data are downloadable at Zenodo repository: https://doi.org/10.5281/zenodo.4637676.

## Ethics Statement

The study followed a protocol approved by the Local Ethics Committee, Comitato Etico per la Sperimentazione con l’Essere Umano della ASL 3 di Genova, in accordance with institutional requirements and national legislation. A written informed consent was obtained for the participation.

## Author Contributions

AB and GB-B conceived, designed the experiment, performed the analysis, and wrote the manuscript. AB collected the data. Both authors contributed to the article and approved the submitted version.

## Conflict of Interest

The authors declare that the research was conducted in the absence of any commercial or financial relationships that could be construed as a potential conflict of interest.
